# DNA damage response and GATA4 signaling in cellular senescence and aging-related pathology

**DOI:** 10.3389/fnagi.2022.933015

**Published:** 2022-09-13

**Authors:** Hao Xiong, Fuzhou Hua, Yao Dong, Yue Lin, Jun Ying, Jie Liu, Xifeng Wang, Lieliang Zhang, Jing Zhang

**Affiliations:** ^1^Department of Anesthesiology, The Second Affiliated Hospital of Nanchang University, Nanchang, China; ^2^Key Laboratory of Anesthesiology of Jiangxi Province, Nanchang, China; ^3^Department of Anesthesiology, The First Affiliated Hospital of Nanchang University, Nanchang, China

**Keywords:** DNA damage response, CGATA4, cellular senescence, atherosclerosis, heart failure

## Abstract

Aging is the continuous degradation of biological function and structure with time, and cellular senescence lies at its core. DNA damage response (DDR) can activate Ataxia telangiectasia-mutated serine/threonine kinase (ATM) and Rad3-related serine/threonine kinase (ATR), after which p53 activates p21, stopping the cell cycle and inducing cell senescence. GATA4 is a transcription factor that plays an important role in the development of many organs, such as the heart, testis, ovary, foregut, liver, and ventral pancreas. Studies have shown that GATA4 can also contribute to the DDR, leading to aging. Consistently, there is also evidence that the GATA4 signaling pathway is associated with aging-related diseases, including atherosclerosis and heart failure. This paper reviews the relationship between GATA4, DDR, and cellular senescence, as well as its effect on aging-related diseases.

## Introduction

Aging is the inevitable spontaneous deterioration of living organisms over time. It is a complex natural phenomenon, manifesting as structural degeneration and functional decline, as well as decreased adaptability and resistance (Davalli et al., 2016). The aging process damages the function of the human body at many levels, resulting in the gradual decline of its ability to resist stress, injury, and disease. Cell senescence is a stable state of cell cycle arrest. Even if the best growth environment and mitotic stimulation are provided, senescent cells still cannot proliferate. Many different triggers can lead to cell aging, including telomere dysfunction, DNA damage, organelle stress, and oncogene activation (Di Micco et al., 2021). Although it leads to the slow deterioration of the organism over long periods, cellular senescence has many physiological functions, including tissue repair, tumor inhibition, organic aging, and control of embryogenesis. DNA damage is arguably the strongest inducer of cell aging because DNA contains the information of all proteins and RNA produced by cells (Ou and Schumacher, 2018).

DNA damage can activate DDR, a signaling pathway that leads to cellular senescence. GATA proteins are a family of factors that play a role in the development of organisms, including six members (GATA1 to GATA6) (Viger et al., 2008). Gata1/2/3 plays a role in hematopoietic cells (Orkin, 1992). Gata4/5/6 protein mainly exists in mesoderm and endoderm-derived tissues, such as the heart and gonads (Molkentin, 2000). In the GATA family, only GATA4 is involved in activating aging, and other family members are not involved (Kang et al., 2015). GATA4 is a transcription factor that regulates signal response processes in many organs, such as cardiac precursor cell differentiation, cardiac development, cardiac hypertrophy, and resistance to apoptosis, as well as mediating the effects of genetic mutations caused by congenital heart disease. GATA4 regulates proteins in a context-dependent manner, thereby performing multiple functions. It has been reported that GATA4 is regulated upstream by the DDR pathway co-opting ATM and ATR, and downstream in a manner different from the conventional DDR pathway, leading to senescence. In addition, alterations in the GATA4 signaling pathway have been frequently observed in various age-related diseases, including atherosclerosis (AS) and heart failure (HF).

This paper reviews the two traditional pathways of DDR, and introduces GATA4 as an alternative player to DDR, followed by a discussion of the regulatory mechanism of GATA4 and its role in AS and HF.

## DNA damage and cellular senescence

Cellular senescence is a process in which the ability of cell proliferation, differentiation, and physiological function gradually declines with time. It was first observed by Hayflick and Moorhead in the 1960s that after a limited number of cell divisions, normal cultured human fibroblasts entered an irreversible cell cycle arrest period called replicative senescence (Hayflick and Moorhead, 1961). Inevitably, this led to discussions about the function of aging cells. There is no doubt that senescent cells build up as time goes by, leading to aging and aging-related diseases, such as AS (Childs et al., 2016), osteoarthritis (Price et al., 2002), or type 2 diabetes (He and Sharpless, 2017). However, cell senescence can also prevent malignant transformation and inhibit the development of cancer. Although senescent cells are involved in the formation of aging-related diseases, they also play an important role in certain physiological processes, such as wound healing (Demaria et al., 2014), embryogenesis, tissue remodeling, and tissue repair (Davaapil et al., 2017). These functions have led to the theory of antagonistic pleiotropy, which postulates that selection can have a beneficial effect early in life, and produce unselected harmful effects in aging organisms (Campisi, 2003). Although this idea remains controversial (Giaimo and D'adda Di Fagagna, 2012), it has led to speculation that senescent cells are beneficial to the body in the early stage, and will be harmful to the body as the accumulation of senescent cells passes a certain threshold.

Cell senescence can be induced in response to various stressors, including telomere dysfunction, DNA damage, organelle stress, and oncogene activation (Di Micco et al., 2021). However, all these stresses share nuclear DNA damage as a common factor, which may be caused by replication stress and endogenous metabolic reactions or exogenous sources, such as chemotherapy and radiation (Mehta and Haber, 2014). There are several different types of damage: intra- and interstrand cross-links, base lesions, single- or double-strand breaks (DSBs), and DNA–protein cross-links (Lindahl, 1993). Compared with other types, DSBs appear less often but are more difficult to repair and the damage is more serious, so this is often a problem worthy of attention. DSBs can activate DDR, a signal transduction pathway that is activated by replication stress and DNA damage. The DDR pathway supplies a mechanism for transmitting signals from sensors, which are transmitted to a series of downstream effector molecules through a transduction cascade to identify the damage, safeguard cells, and remove the threat to the organism (Harper and Elledge, 2007; Hartlerode and Scully, 2009).

The exact nature of DNA damage receptors at the beginning of the DDR pathway is still unknown. Since DNA damage can activate DNA-dependent protein kinase (DNA-PK) and poly (ADP-ribose) polymerase (PARP), these proteins have commonly been regarded as DNA damage sensors, although they are not always involved (Wang et al., 1995; Jimenez et al., 1999). DNA-PK, which consists of DNA terminal binding protein Ku and a catalytic subunit, can increase the DNA-dependent phosphorylation of some proteins (Gottlieb and Jackson, 1993). Ku is a heterodimer, composed of 70 and 80 kDa subunits. However, we do not know much about the specific structure of the Ku subunits (Wu and Lieber, 1996). When DNA-PK is located in a DSB, the DDR signal is activated and transmitted to the downstream protein kinase signal cascade. ADP ribosylation is a modification that can be mediated by 16 family members, but only PARP1 and PARP2 are related to DDR. PARP1 and PARP2 are ADP ribose polymerases and regulate stress response processes and DNA repair. They are activated by DNA damage and recruit DDR factors by adding ADP-ribose chains to proteins (Schreiber et al., 2006).

Compared with the understanding of damage sensors, we have a better understanding of transducers, among which ATM and ATR play a key role. These proteins belong to the phosphatidylinositol 3-kinase-like protein kinase (PIKK) family. ATM mutations are found in human diseases and have been investigated in some detail. DNA damage directly or indirectly stimulates ATM activity, which in turn activates proteins, such as BRCA1, Chk2, Nbs1, p53, and Mdm2 to respond to DNA damage. By contrast, there are few known ATR mutations in human diseases, and less is known about this protein. ATR mainly mediates ATM-independent phosphorylation events. There is evidence that the DNA damage signal pathways of ATM and ATR are parallel but partially overlapping, while mainly responding to different types of damage.

There is evidence that ATM and ATR partially regulate CHK1 and CHK2 kinases downstream of the DDR response pathway. CHK1 and CHK2 are two serine/ threonine kinases with different structures but partially overlapping substrates. CDc25A and CDc25C phosphatases are phosphorylated by activated CHK2, resulting in their inactivation or degradation. The active CDC25A/C eliminates the inhibitory phosphorylation on CDKs that drive cell cycle progression, resulting in cell cycle arrest. CHK2 phosphorylates tumor inhibitor p53 at the ser15 and ser20 residues, leading to its stabilization and activation, finally resulting in cell cycle arrest (Ou et al., 2005). The ATM-CHK2-P53 pathway controls the G1 checkpoint. ATR activates CHK1 through phosphorylation at serine 345, resulting in CHK1 autophosphorylation at serine 296 to achieve complete activation. Similar to CHK2, CHK1 phosphorylates and thereby inactivates CDC25A/C (Lewis et al., 2017; Rundle et al., 2017). In yeast, phosphorylated CHK1 activates and then phosphorylates Wee1 kinase, thereby deactivating CDK1 and 2 (Lee et al., 2001; Lin et al., 2017). The ATR-CHK1-WEE1 pathway controls the S and G2/M checkpoints. It has been found that ATR-Chk1 plays a major role in replication checkpoints and DNA damage, and ATM-Chk2 plays a secondary role in the response to DNA double-strand breaks (Bartek and Lukas, 2003; Kastan and Bartek, 2004). The ATM-CHK2-Cdc25A pathway or the ATR-Chk1-Cdc25A pathway can cause cell cycle arrest by activating p53, which is a transcription factor that controls genes related to DNA repair, cell cycle arrest, apoptosis, and metabolism (Fischer, 2017). The phosphorylation of p53 leads to an increase in p53 levels (Zhang and Xiong, 2001). P53 activates the expression of cyclin-dependent kinase inhibitor p21, a component of the tumor suppressor pathway that enables p53 control, and an important mediator of aging-related cell cycle arrest. The stable expression of p21 is sufficient to establish and maintain senescence-related growth arrest. P16 is also a cyclin-dependent kinase inhibitor, which is part of the RB-controlled tumor inhibitor pathway. The activation of p16 can inhibit cyclin d-cdk4/6. Both p21 and p16 can maintain the active form of retinoblastoma protein (PRB), leading to cell aging and subsequent effects (Campisi and D'adda Di Fagagna, 2007).

### GATA4, a new aging regulator in DDR

GATA factors are a group of transcriptional regulators that play an important role in the development and differentiation of all eukaryotes. GATA4 is a zinc-finger protein that plays an important role in the development of many organs, such as the heart, testis, ovary, foregut, liver, and ventral pancreas (Viger et al., 2008). However, in 2015, Kang et al. found that GATA4 is activated by ATM and ATR in DDR, through an axis that is different from the p53 and p16^INK4a^ pathways, and then activates nuclear factor kappa B (NF-κB), leading to the formation of senescence-associated secretory phenotype (SASP), illustrating the important role of GATA4 in cell aging for the first time (Kang et al., 2015).

It is generally believed that there are two main pathways of cell senescence caused by DDR. The first is related to p53, which is activated by DDR injury signals (Roos and Kaina, 2013), and then activates p21 (Abbas and Dutta, 2009), which further leads to cell cycle arrest (Deng et al., 1995). The other is related to p16, which negatively regulates the pRB/E2F pathway and continuously activates pRB, preventing E2F transcription factors from interacting with multiple genes involved in proliferation (Sherr and McCormick, 2002). Both pathways eventually stop the cell cycle (Barnes et al., 2019).

However, Kang et al. found a new pathway of DDR leading to aging, in which GATA4 leads to cell aging by regulating SASP, not by stopping the cell cycle. The study was well designed. By examining the substances that promote the expression of miR-146a in aging cells, GATA4 was found. The function of GATA4 in regulating aging the phenotype seems to be unrelated to the p53 and p16INK4a/RB pathways. It was concluded that the GATA4 pathway regulates SASP, which seems to be an independent pathway of DDR (Kang et al., 2015).

### Relationship between autophagy, cell senescence, and GATA4

Autophagy is a process in which cells engulf cytoplasmic proteins or organelles into vesicles, which then fuse with lysosomes to form autophagic lysosomes and degrade their encapsulated contents, to realize the metabolic needs of cells and the renewal of organelles. Autophagy is a dynamic process that includes a double-layer membrane budding from the ribosome-free area of the rough endoplasmic reticulum, which then wraps a part of the cytoplasm, organelles, and proteins that need to be degraded in the cell to form autophagosomes, which are fused with lysosomes to form autophagic lysosomes and degrade the contents. Autophagy is another key effector program of cell aging, although it was initially considered to primarily play a role in the hunger response. It recovers cellular components and recycles them into cellular material metabolism to maintain energy homeostasis (Kang et al., 2007; Kang and Avery, 2008, 2009; Kroemer et al., 2010; Boya et al., 2013; Klionsky and Codogno, 2013). However, autophagy can be activated by many stresses during cell aging, and autophagy was indeed found to be increased in aging cells. However, its role in cell senescence is still under discussion (Kuilman et al., 2010; Gewirtz, 2013; Kang and Elledge, 2016). On the one hand, autophagy is thought to promote cellular senescence, and it was generally believed that autophagy inhibits cellular senescence only through its homeostatic role. However, it was found that the removal of several key autophagy regulators retards cellular senescence, which led to the hypothesis that autophagy provides the basis for SASP protein synthesis (Young et al., 2009). A special type of autophagy responsible for protein synthesis of some SASP factors, as a trans autophagy spatial coupling compartment, was later discovered (Narita et al., 2011). Autophagy generates amino acids and other metabolic substances, which are then used for the bulk synthesis of SASP proteins that promote senescence. On the other hand, it was generally believed that autophagy clears stressors that contribute to aging, leading to the proposal that autophagy protects against aging (Kroemer et al., 2010; Rubinsztein et al., 2011; Boya et al., 2013). Autophagy can decrease the level of oxidative stress, and enhancing the cell's autophagy ability under oxidative stress can inhibit cell senescence (Han et al., 2016; Tai et al., 2017). Additionally, mitochondrial dysfunction is a hallmark of cellular senescence, and elevated superoxide levels could promote mitochondrial dysfunction (Lopez-Otin et al., 2013). Autophagy can also clear dysfunctional mitochondria, and selective autophagy of mitochondria in senescent cells reduces several features of senescence (Ito et al., 2015; Korolchuk et al., 2017).

Transcription factor GATA4, which plays an important role in SASP and cell aging regulation, was identified when studying the influencing factors of cell aging. GATA4 protein, but not its mRNA, accumulates during cell senescence due to increased protein stability. With the progress of research, it was found that the stability of GATA4 protein was regulated by autophagy. In general, autophagy receptor protein SQSTM1/p62 binds to GATA4 and degrades it through autophagy. However, when cells are exposed to aging-related stimuli, the binding of SQSTM1/p62 to GATA4 will be reduced, GATA4 will not be degraded by autophagy, and the accumulated GATA4 will activate SASP and cell aging. Aging-induced stimulation leads to general autophagy, which may be completed by the trans autophagy spatial coupling compartment (TASCC) to promote SASP and cell aging. This view can be proved by regulating the time of autophagy. Transient inhibition of autophagy leads to aging more effectively than continuous inhibition. Cell aging induced by the consumption of sqstm1 is more effective than the consumption of core autophagy regulators ATG7 or ATG5 under normal conditions, which further supports this view. Moreover, some studies have found that selective autophagy is first started under aging-related stimulation. Selective autophagy can inhibit cell aging and protect cells from aging through the degradation of aging regulator GATA4. Subsequently, general autophagy begins that promotes aging through TASCC. Therefore, autophagy has a dual effect on aging.

### GATA4 in the DNA damage response

Autophagy regulates GATA4 through autophagy receptor protein SQSTM1/p62, but little is known about the mechanisms of the interaction between GATA4 and SQSTM1. However, it was found that DDR initiates the pathway of GATA4-regulated aging and activates the kinases ATM and ATR, both of which inhibit p62. These data suggest that ATM and ATR control the selective autophagy of GATA4. The affinity between SQSTM1 and its substrate is affected by the phosphorylation of SQSTM1. Therefore, it is possible to explore whether ATM and ATR reduce their interaction by phosphorylating SQSTM1 or GATA4. It was also proposed that ATM and ATR may act as signal sensors that can adjust the interaction between GATA4 and SQSTM1. The latter mainly recognizes ubiquitinated substrates, which can be used as a signal sensor to add GATA4 and SQSTM1 to the interaction. Perhaps some other substrates of SQSTM1 may increase due to stimulation by aging-activated ATM and ATR, and these may have a higher binding affinity for SQSTM1 than GATA4. This question should be addressed in the future because it will enable us to further understand the relationship between autophagy and cell aging, as well as the specific mechanism through which the DDR regulates GATA4.

It was established by Ran Jansen 30 years ago that ATM and ATR can inhibit p62 and reduce the degradation of GATA4, which in turn can activate NF-κB (Sen and Baltimore, 1986). NF-κB is present in almost all animal cell types and is involved in cell responses to diverse stimuli including, cytokines, free radicals, heavy metals, UV irradiation, oxidized low-density lipoprotein (LDL), and bacterial or viral antigens. It also regulates tumor progression, innate immunity, the inflammatory response, and acquired immune processes (Basseres and Baldwin, 2006). There are five members of the NF-κB family, including NF-κB1 (P50), NF-κB2 (p52), RelA (p65), RelB, and C-Rel (Hayden and Ghosh, 2008). The NF-κB family proteins contain a highly conserved N-terminal Rel homology domain (RHD), which is a conserved sequence of approximately 300 amino acids with dimerization sequence-specific DNA binding and inhibitory protein binding functions (Hayden and Ghosh, 2012; Smale, 2012). NF-κB members can be divided into p50 or p52 groups, which are respectively produced by the cleavage of p110 and p105 precursors, and the C-terminal region of connexin repeat (AnkR) is cleaved after translation from p50 and p52. RelA (p65), RelB, and C-Rel are synthesized as mature proteins with transcriptional transactivation domains (TADS) (Smale, 2012). Generally speaking, NF-κB refers to NF-κB1 dimer protein formed by p65/p50 subunits. By contrast, the RelB/p52 subunits form NF-κB2 dimer protein, although there are other combinations with diverse functions, including p50:p50 and p52:p52 homodimers or p52:RELA and p50:RELB heterodimers (Adler et al., 2008; Smale, 2012).

There is evidence that the NF-κB transcription factors can activate SASP (Chien et al., 2011; Crescenzi et al., 2011; Freund et al., 2011; Rovillain et al., 2011). Chien et al. established an aging model with human IMR-90 skin fibroblasts and it was observed that NF-κB promoted the formation of SASP, whereby the p65 subunit of NF-κB accumulated in aging cells in the model. NF-κB controlled a larger and different transcription module than p53 and Rb, influencing the non-cellular autonomous aspects of the aging process (Chien et al., 2011). SASP was first defined in 2008. Judith Campisi and her team found that aging cells induced by genotoxic stress secreted many substances related to inflammation and carcinogenesis, and this phenotype was defined as senescence-associated secretory phenotype (SASP) (Coppe et al., 2008). SASP is characterized by transcriptional activation of cytokines, chemokines, growth factors, and extracellular matrix (ECM) proteases, which may promote the aging of the secreting cells themselves, change the microenvironment of the surrounding tissues of aging cells, and finally affect the whole body. SASP is a powerful secretory program, including hundreds of biological proteins and non-protein active factors, including hemostatic factors, proteases, bradykinins, ceramides, damage-related molecular patterns, and ECM components (Davalos et al., 2013; Wiley et al., 2019; Basisty et al., 2020). Different tissues experience aging and the different inducing factors of aging. There are some qualitative and quantitative differences between the SASP of different cells, but there is the main group of secreted factors in all types of aging cells *in vitro*, including CXC chemokine ligand 8 (CXCL8), inflammatory interleukin-6 (IL-6), and monocyte chemoattractant protein 1 (MCP1) (Coppe et al., 2008). In addition to pro-inflammatory molecules, SASP also includes enzymes that regulate ECM remodelings, such as serine/cysteine protease inhibitors (Eren et al., 2014), matrix metalloproteinases (MMPs) (Coppe et al., 2010), and metalloproteinase tissue inhibitors (Özcan et al., 2016). Cells affected by SASP release many factors, including transforming growth factor-β (TGF-β) family members, vascular endothelial growth factor (VEGF), as well as chemokines such as CCL2 and CCL20 (Acosta et al., 2013). The aging caused by the release of these factors is called paracrine senescence. TGF-β can induce cyclin-dependent kinase inhibitors p15INK4B, p21, and p27, as well as inhibit c-myc (Zhang et al., 2017) and a variety of proliferation factors to promote cell aging. TGF-β can induce mitochondria to produce reactive oxygen species (ROS) in a variety of cell types, and ROS signals that pass through gap junctions can induce the DNA damage response in adjacent cells (Nelson et al., 2012). Finally, TGF-β also downregulates telomerase reverse transcriptase (TERT) expression and inhibits telomerase activity.

### Associations of GATA4 with aging-related diseases

**Figure 1:**
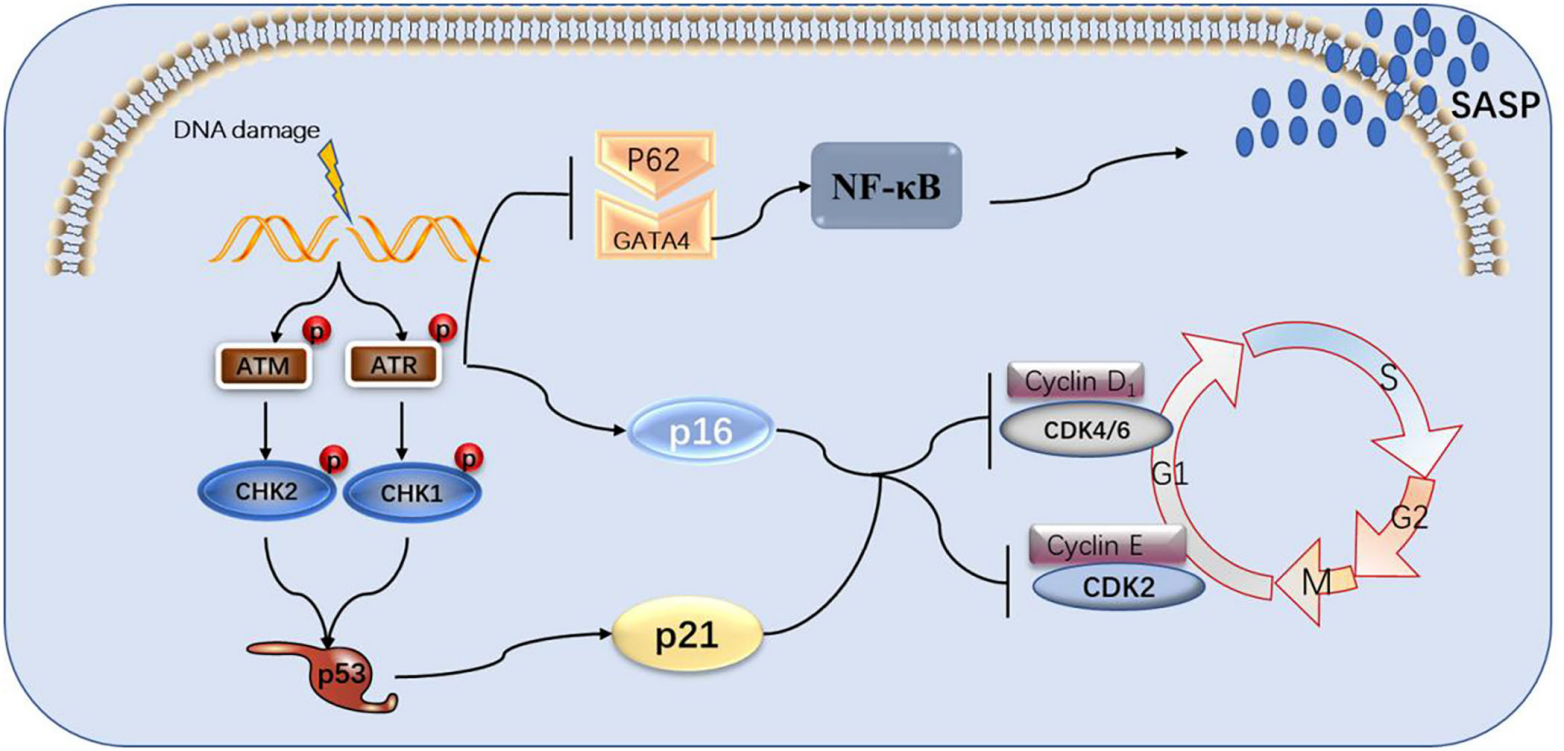
The role of GATA4 in atherosclerosis and myocardial hypertrophy. The GATA4-TWIST1-Snail signaling pathway promotes EC dysfunction and atherosclerosis. GATA4 may regulate cell hypertrophy by increasing the expression level of GATA4, DNA binding activity, and transcriptional activity under the stimulation of the hypertrophy signal.

GATA4 is linked to a pathway through which DDR leads to senescence. GATA4 can not only cause cell senescence but also plays a role in cardiovascular development and disease. For example, overexpression of GATA4 can lead to cardiac hypertrophy and heart failure (Zhou et al., 2012). Furthermore, GATA4 is more expressed in atherosclerotic endothelial cells (Mahmoud et al., 2019) (Figure 1). These diseases are all related to aging, and GATA4 regulates the pathological process of these diseases at the molecular level, playing an important role in the process of disease formation.

## Roles of GATA4 in atherosclerosis

Atherosclerosis (AS) is a disease of aging, a chronic degenerative disease that gradually increases in morbidity and mortality with age. AS is the main cause of coronary heart disease, cerebral infarction, and peripheral vascular disease. The disorder of lipid metabolism is considered the pathological basis of AS. The lesions of the affected arteries begin from the intima, generally followed by the accumulation of lipids and complex sugars, bleeding, and thrombosis, followed by fibrous tissue hyperplasia and calcium deposition, leading to the gradual metamorphosis and calcification of the middle layer of the artery, resulting in the thickening and hardening of the arterial wall and stenosis of the vascular cavity. Lesions often involve large and medium-sized muscular arteries. Once they develop enough to block the arterial cavity, the tissues or organs supplied by the artery will become ischemic or necrotic. Because the appearance of lipids accumulated in the intima of the artery is yellow and sclerotic, it is called AS. There are many causes of AS, including hypertension, hyperlipidemia, and heavy smoking, as well as diabetes, obesity, and genetic factors. Vascular EC plays an important role in maintaining vascular health, and the injury and dysfunction of ECs are the initial factors of AS. ECs are subjected to a mechanical force of axial stress by flowing blood, which is also known as shear stress. In the areas affected by vascular stenosis and bifurcation, the blood flow is disordered and subjected to multidirectional and low shear stress, which increases the probability of AS. Conversely, where the blood flow is stable, the incidence of AS is low under uniform and high shear stress (Kwak et al., 2014). Blood flow disorder can promote EC dysfunction by repeatedly inducing inflammation and EC proliferation, which contributes to the initiation and development of AS (Dai et al., 2004). EC proliferation will lead to leaky junctions between cells, induce the accumulation of LDL and promote the progression of AS (Cancel and Tarbell, 2011). Blood flow can promote endothelial gene expression to promote EC dysfunction (Passerini et al., 2004), but the exact mechanism through which blood flow promotes endothelial gene expression is not fully understood.

GATA4 was also found to be associated with AS. GATA4 and Twist1 are preferentially expressed at the site of AS, where the GATA4-Twist1 signal can induce EC dysfunction to accelerate the development of AS (Mahmoud et al., 2016). However, the specific mechanisms underlying the relationships between GATA4, Twist1, and AS are still unclear. Blood flow disorder will lead to changes in the expression of hundreds of genes. Twist1 and GATA4 also show increased expression in areas with atherosclerotic blood flow disorder. The en face staining of mouse aortic endothelium showed that blood flow disorder increased the contents of Twist1 and GATA4 protein in the bending part of the aortic arch (Serbanovic-Canic et al., 2017). There is evidence that low shear stress promotes the expression of Twist1t and GATA4 (Dardik et al., 2005; Warboys et al., 2014), whereby GATA4 acts upstream of Twist1 and promotes the expression of Twist1 in EC cells. These data suggest that blood flow disorder promotes the expression of Twist1 and GATA4 (Mahmoud et al., 2016). Studies have found that the endothelial-to-mesenchymal transition (EndoMT) plays an important role in the process of AS (Moonen et al., 2015). The GATA4-Twist1 signal activates the transcription factor Snail to drive EndoMT, resulting in EC dysfunction (Mahmoud et al., 2017).

## Roles of GATA4 in heart failure

Heart failure (HF) indicates that the amount of venous returning blood cannot be fully discharged from the heart due to the impairment of the systolic and/or diastolic function, resulting in venous system blood stasis and insufficient arterial system blood perfusion, finally resulting in cardiac circulatory disorder syndrome. This disorder syndrome is mainly manifested as pulmonary and vena cava congestion. HF is not an independent disease, but the final stage of the development of heart disease. The vast majority of HF cases start with left HF, that is, the first manifestation is pulmonary congestion. Myocardial hypertrophy is a pathological process related to HF. The prevalence and incidence of HF increase with age (Hammond and Rich, 2019), and the occurrence of myocardial hypertrophy will increase the risk of HF. Cardiac aging is characterized by cardiac hypertrophy, cardiac fibrosis, and the accumulation of dysfunctional mitochondria. Macroautophagy can remove dysfunctional mitochondria and damaged DNA to improve heart function (Shirakabe et al., 2016). Myocardial hypertrophy is an increase in the thickness of the ventricular wall and/or ventricular septum caused by external mechanical stimulation or neurohumoral factors such as angiotensin II, endothelin, or catecholamine.

There is evidence that GATA4 is related to myocardial hypertrophy, and overexpression of GATA4 induces myocardial hypertrophy in experimental models (Liang et al., 2001). However, little is still known about the specific mechanism of cell hypertrophy. The hypertrophy stimulation signal leads to the increase of GATA4 transcriptional activity through four signaling pathways. The first is the extracellular signal-regulated kinase (MEK1-ERK1/2) signal pathway. Hypertrophy stimulation can stimulate ERK2 to phosphorylate serine residues of GATA4, resulting in an increase in DNA binding activity and GATA4 transcriptional activity (Ueyama et al., 2000). The second is the Rho/ROCK pathway, in which Rock1 can cause ERK phosphorylation and activate GATA4 through the MEK1-ERK1/2 pathway (Yanazume et al., 2002). The third is the mitogen-activated protein kinase (MAPK) pathway. Some studies have that there are MAPK-targeted phosphorylation sites in the N-terminal transactivation domain of GATA4, which may be involved in the signal transduction process of MAPK during myocardial hypertrophy (Tenhunen et al., 2004). The last one is the signal transduction pathway dependent on activated T nuclear factor transcription factor and calcineurin (CAN/NFAT3/GATA4). In this pathway, hypertrophy stimulation will increase plasma calcium levels and activate can dephosphorylate NFAT3, after which the dephosphorylated NFAT3 enters the nucleus and interacts with GATA4 to activate multiple genes related to myocardial hypertrophy (Vega et al., 2003). Recently, it has also been found that deacetylation of GATA4 has an anti-myocardial hypertrophy effect (Yamamura et al., 2020).

## Conclusion and perspectives

**Figure 2:**
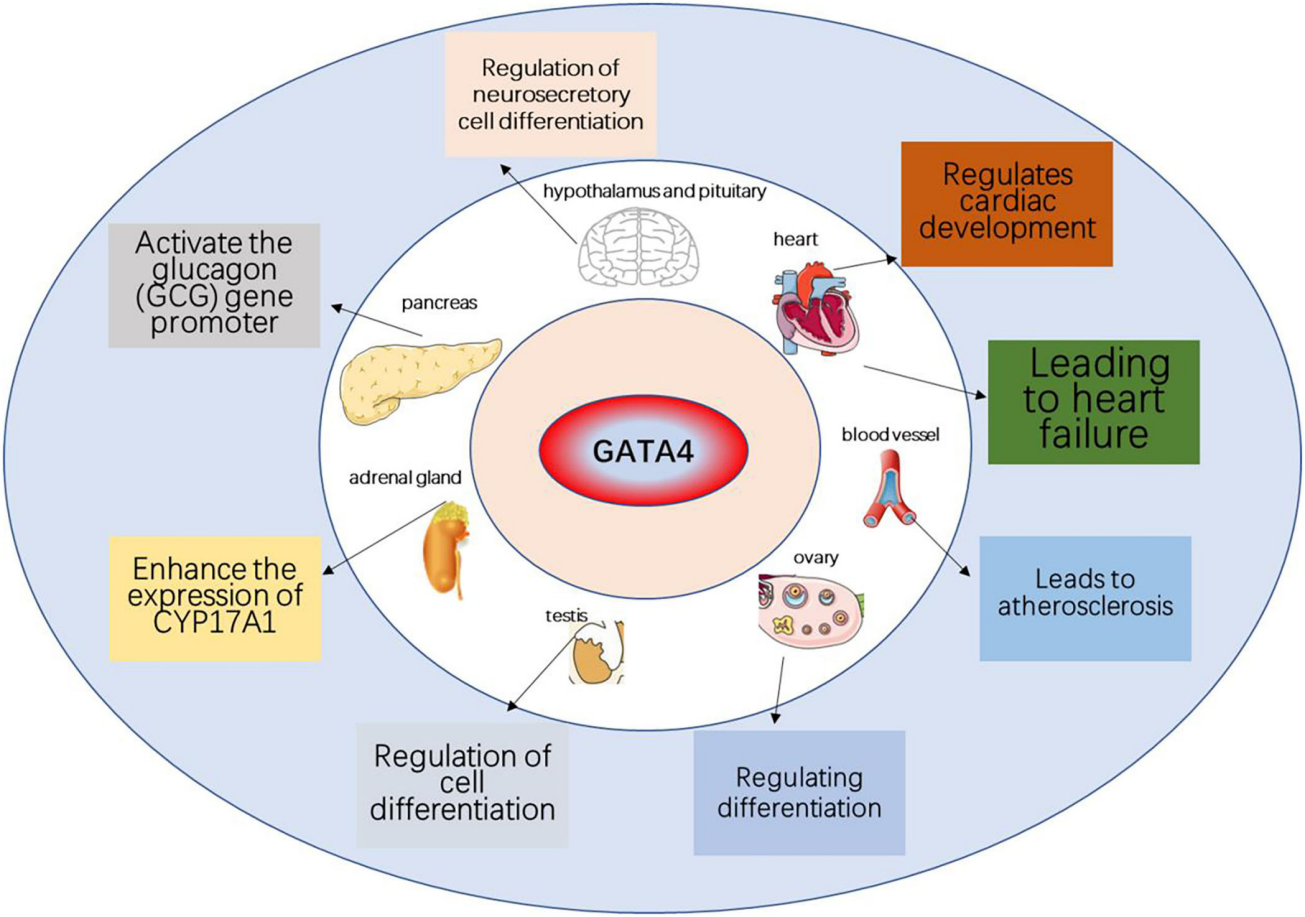
The process of DNA damage leading to cell senescence and the role of GATA4 in it. DNA damage signals activate ATM and ATR, causing the p53 and p16 pathways to activate, blocking cell cycles and, in turn, aging cells. ATM and ATR can also suppress selective autophagy between p62 and GATA4, activating NF-κB and SASP.

**Figure 3:**
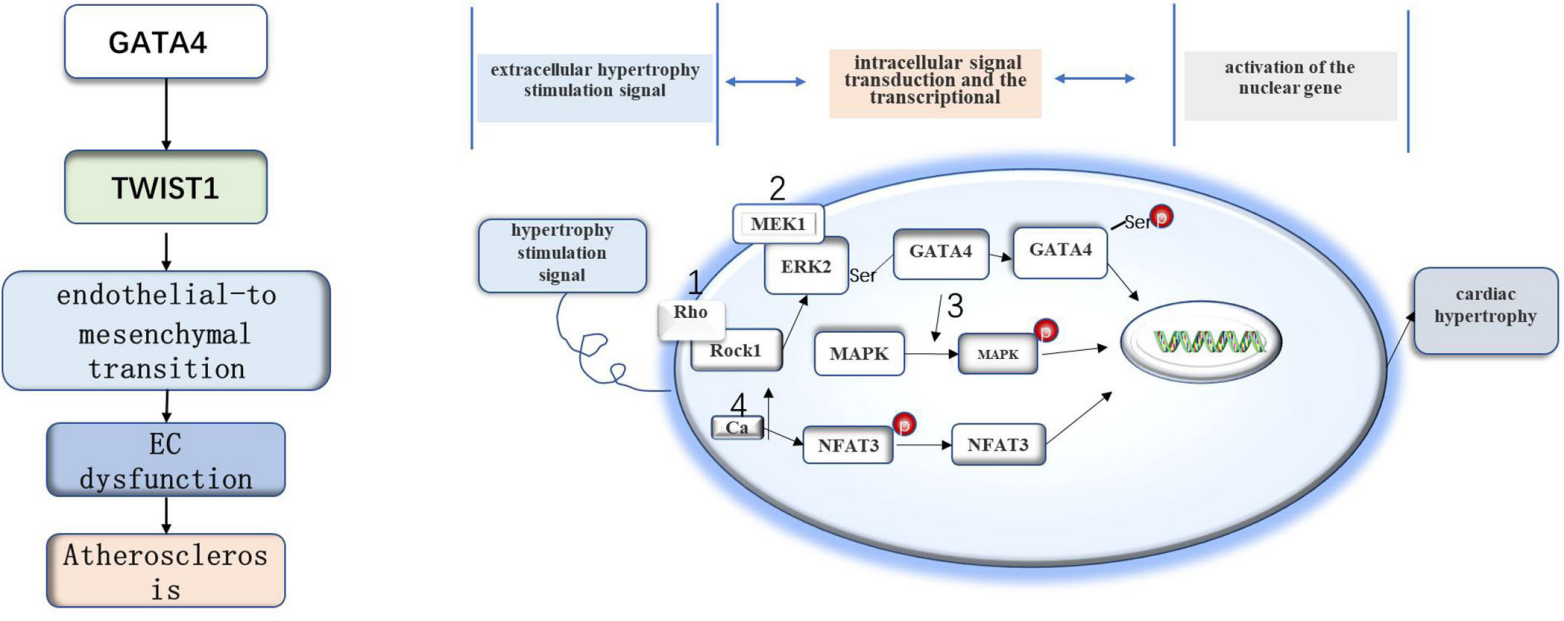
The role of GATA4 in various organs and aging-related diseases. GATA4 can promote the development of heart, testis, ovary, foregut, liver, and ventral pancreas and can lead to heart disease associated with aging.

This paper reviews the mechanisms through which the DDR signaling pathway leads to cellular senescence, the involvement of the GATA4 factor in these processes, as well as the link to atherosclerosis and heart failure. DDR activates ATM and ATR, thereby activating p53 and p16, which activate cell cycle inhibitors, leading to cell cycle arrest and cellular senescence. GATA4 is also regulated by ATM and ATR, which inhibit the binding of p62 and GATA4 to inhibit selective lineage autophagy of GATA4, thereby activating NF-κB and SASP, leading to cellular senescence (Figure 2). Senescent cells release large amounts of secreted substances that affect the microenvironment around the cells and may lead to senescence of other surrounding cells. The accumulation of senescent cells will lead to the destruction and diminished function of tissues, organs, and systems, which will eventually lead to the aging of organisms and aging-related diseases. GATA4 plays a role in the development of several human organs, but it also causes endothelial dysfunction, promoting atherosclerosis and hypertrophy of the heart muscle, leading to heart failure (Figure 3).

This paper reviewed the known roles of GATA4 in the DNA damage response, cell aging, and aging-related diseases, this provides many possibilities, that is, to invent a drug to promote the autophagy of GATA4 and block the transmission of GATA- NF-κB -SASP axis to prevent cell aging, but there are still many problems to be solved. For example, little is known about the mechanism of the interaction between GATA4 and SQSTM1. Their roles span aging, autophagy, inflammation, and SASP. Further understanding the relationship between GATA4 and aging will help us provide a basis for the treatment of cell aging. However, the specific mechanism through which GATA4 activates NF-κB is unclear, and understanding it can also help in regulating cell aging. Further studies of the relationship between GATA4 and aging will help provide a basis for the treatment of cellular aging. In addition, little is known about the specific mechanisms underlying the interplay between GATA4, Twist1, and AS. Understanding the underlying mechanisms may provide new ideas on how to prevent the occurrence and progression of AS. Solving these problems will help to deepen the understanding of GATA4, enable the pharmacological management of GATA4-related biological processes, and greatly contribute to maintaining human health. Targeting GATA4 is expected to provide new treatments for AS and other cardiovascular diseases.

## Author contributions

HX wrote the manuscript. FH provided topic selection. FH, YD, YL, JY, JL, XW, LZ, and JZ provided article revision and guidance support. All authors contributed to the article and approved the submitted version.

## Funding

This work was supported by grants from the National Natural Science Foundation of China (82060219 and 81760261), Natural Science Foundation of Jiangxi Province (2018ZDG40028), Jiangxi Province Thousands of Plans (jxsq2019201023), and Youth Team Project of the Second Affiliated Hospital of Nanchang University (2019YNTD12003).

## Conflict of interest

The authors declare that the research was conducted in the absence of any commercial or financial relationships that could be construed as a potential conflict of interest.

## Publisher's note

All claims expressed in this article are solely those of the authors and do not necessarily represent those of their affiliated organizations, or those of the publisher, the editors and the reviewers. Any product that may be evaluated in this article, or claim that may be made by its manufacturer, is not guaranteed or endorsed by the publisher.

## Abbreviations

DDR: DNA respond damage
ATM: Ataxia telangiectasia mutated serine/threonine kinase
ATR: Rad3-related serine/threonine kinase
CDK: Cyclin dependent kinase
TRAF3IP2: Tumor necrosis factor receptor-related factor interacting protein 2
IL1A: Interleukin 1a
DSBs: Double-strand breaks
DNA-PK: DNA-dependent protein kinase
PARP: Poly (ADP-ribose) polymerase
PIKKs: Phosphatidylinositol 3-kinase-like protein kinase
CHK: Checkpoint kinase
TASCC: Trans autophagy spatial coupling compartment
NF-κB: Nuclear factor activated B cells
ECM: Extracellular matrix
CXCL8: CXC chemokine ligand 8
IL-6: Inflammatory interleukin-6
MCP1: Monocyte chemoattractant protein 1
MMPs: Matrix metalloproteinases
TGF- β: Transforming growth factor-β
VEGF: Vascular endothelial growth factor
ROS: Reactive oxygen species
TERT: Telomerase reverse transcriptase
AS: Atherosclerosis
EC: Endothelial cells
LDL: Low density lipoprotein
HF: Heart failure
MAPK: Mitogen-activated protein kinase system.
